# Rapid One-Pot Microwave Assisted Green Synthesis Nitrogen Doped Carbon Quantum Dots as Fluorescent Precursor for Estimation of Modafinil as Post-Covid Neurological Drug in Human Plasma with Greenness Assessments

**DOI:** 10.1007/s10895-022-03128-5

**Published:** 2022-12-28

**Authors:** Baher I. Salman, Ahmed I. Hassan, Yasser F. Hassan, Roshdy E. Saraya, Hany A. Batakoushy

**Affiliations:** 1 Pharmaceutical Analytical Chemistry Department, Faculty of Pharmacy, Al-Azhar University, Assiut Branch, Assiut, 71524 Egypt; 2 Pharmaceutical Analytical Chemistry Department, Faculty of Pharmacy, Port Said University, Port Said, 42511 Egypt; 3 Pharmaceutical Analytical Chemistry Department, Faculty of Pharmacy, Menoufia University, Shebin Elkom, 32511 Egypt

**Keywords:** Modafinil, Fluorescence, Human plasma, Dosage form, N@CQDs

## Abstract

The neuro-stimulant anti-narcoleptic drug as modafinil (MOD) is used to treatment neurological conditions caused by COVID-19. MOD was used to treatment narcolepsy, shift-work sleep disorder, and obstructive sleep apnea-related sleepiness. So, an innovative, quick, economical, selective, and ecologically friendly procedure was carried out. A highly sensitive N@CQDs technique was created from green Eruca sativa leaves in about 4 min using microwave synthesis at 700 w. The quantum yield of the synthesized N@CQDs was found to be 41.39%. By increasing the concentration of MOD, the quantum dots' fluorescence intensity was gradually quenched. After being excited at 445 nm, the fluorescence reading was recorded at 515 nm. The linear range was found to be in the range 50 – 700 ng mL^−1^ with lower limit of quantitation (LOQ) equal to 45.00 ng mL^−1^. The current method was fully validated and bio analytically according to (US-FDA and ICH) guidelines. Full characterization of the N@CQDs has been conducted by high resolution transmission electron microscope (HRTEM), Zeta potential measurement, fluorescence, UV–VIS, and FTIR spectroscopy. Various experimental variables including pH, QDs concentration and the reaction time were optimized. The proposed study is simply implemented for the therapeutic drug monitoring system (TDMS) and various clinical laboratories for further pharmacokinetic research.

**Supplementary Information:**

The online version contains supplementary material available at 10.1007/s10895-022-03128-5.

## Introduction

A neurological condition known as narcolepsy is characterized by an inability to control sleep–wake cycles. Narcoleptics may fall asleep at inappropriate times and experience daytime fatigue [1].

**Fig. 1:**
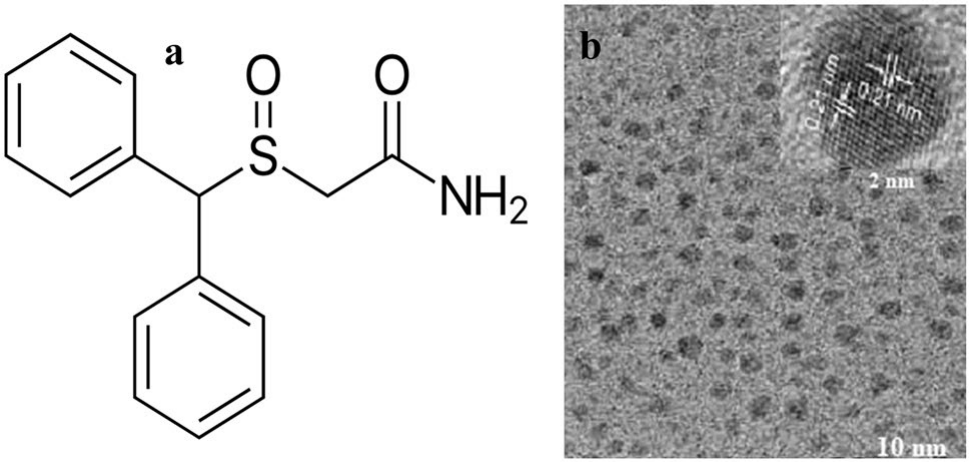
**a** Chemical structure of MOD, and **b** HR-TEM image of carbon dots

**Modafinil** (MOD, Fig. 1a) is (2-[(diphenylmethyl)sulfinyl] acetamide), MOD is being approved to treat narcolepsy, obstructive sleep apnea, and shift work sleep disorder, MOD is a special CNS stimulant, it differs from other central nervous system stimulants therapeutically and pharmaceutically in that it generates long-lasting waking effects without behavioral alteration, addictive qualities, or sleep rebound [1].

Various analytical methods were reported for estimation of MOD as HPLC [1–6], UPLC [7], HPTLC [8], fluorimetric [9], spectrophotometry [10, 11] and capillary electrophoresis [12].

In the present study, novel, simple, fast synthesis, environmentally friendly, organic solvents free, ultra-sensitive spectrofluorimetric approach using nitrogen from natural source to be doped in quantum dots. The proposed method (N@CQDs) provides ultra-sensitive (50 – 700 ng mL^−1^) and it was selective for estimation of MOD in pharmaceutical dosage form, content uniformity and human plasma than other reported fluorimetric method (0.5 – 20 µg mL^−1^) [9]. The N@CQDs easily and rapid synthesized from green source using microwave synthesis in 4 min with high quantum yield 41.39%.

Carbon quantum dots (CQDs), which have distinct optical properties, great water solubility, biocompatibility, non-toxicity, and simplicity of functionalization, have become well-established as an effective analytical sensor in the last ten years. Due to their remarkable and customizable fluorescence properties, CQDs are presently positioned as an excellent replacement for fluorescent dyes and fluorescence derivatizing agents. They are also less harmful to the environment and less poisonous than nanomaterials made of metal [13–15].

Furthermore, different techniques were utilized for synthesis of CQDs as dry heat, hydrothermal and solvothermal methods. However, these methods have various drawbacks as go against the idea of "green chemistry," as they can take up to 24 h, need temperatures up to 300 degrees, and use harsh chemicals and organic solvents. [16] Microwave synthesis quantum dots is a new approach recently applied to reduce the synthesis time form hours to minutes and greenness synthesis with quantum yield product. [17, 18] *Eruca Sativa* leaves is very cheap plant widely growing in Egypt. It is the oldest leaf vegetables consumed by humans. *Eruca Sativa* leaves are rich with various components and vitamins as carbohydrates, sugar, fibers, vitamin A, vitamin B_1_, riboflavin and folic acid*.*

The proposed approach aims to create an integrate strategy for greening both the synthesis process and the carbon source in this work in order to recover the greenness and sustainability of the synthesis of CQDs. Only low-power microwave-assisted synthesis at 700 W for 4 min is used in the suggested technique. We also had access to inexpensive, abundant, and practical plants. Additionally, this study's greenness is consistent with global claims about green chemistry and safety.

## Experimental

### Materials and Reagents

Modafinil (MOD 99.98%) authentic powder was obtained from Mash Premiere, Region 6, Second District, 90 Street, Fifth Settlement, New Cairo City, Egypt. Bravamax^®^ (200 mg tablets) was purchased from the local market, Egypt. Human plasma samples were obtained from Egyptian Blood Bank and stored at -24 °C until analysis.

Standard solution of MOD (100 µg mL^−1^) was prepared using via 10 mg of MOD was dissolving 100 mL methanol.

### Equipment’s of the N@CQDs Method

The results were acquired by an FS5 spectrofluorometer (Edinburgh, UK) with a 150 W xenon lamp source for excitation. Also, with 1-cm quartz cell and connected to Fluoracle^®^ software. The slit widths were set to 2 nm and the scanning speed 1000 nm/min. The dynamic light scattering measurements (DLS) were scanned by Zetasizer Red badge instrument of ZEN 3600 (Malvern, UK). MFMI-100A (MED Future) Microwave instrument (2450 MHz, 0- 1000 W) was designed for catalyzing organic synthesis and solvent extraction. Magnetic and Mechanical stirring- IR Temperature Sensor (0–300'C). Fourier-transform infrared (FTIR) Germany. pH-meter (China). The powder X-ray diffraction (PXRD) was scanned by Philips X-ray diffractometer. High-resolution transmission electron microscope (HR-TEM) images were captured via JEOL JEM-100CX II unit tungsten EM filament 120 (USA).

### Synthesis of Environmentally Green Quantum Dots (N@CQDs)

The green nitrogen carbon quantum dots (N@CQDs) were synthesized using thermolysis of *Eruca Sativa* leaves. The leaves were crushed well and filtrate, then 40 mL of the filtrate was transferred into reaction vessel and then placed in microwave. Microwave source: 2450 MHz, 0- 1000 W for 4 min until brown solution was formed. The residue was dispersed and then sonicated for 30 min to remove large particles. The solution was filtered and centrifuged at 4000 rpm for 10 min. the supernatant was filtrated via 0.45 μm cellulose membrane. The obtained yellow filtrate color solution was utilized for experiment.

### Fluorimetric Analysis of MOD

One milliliter of N@CQDs (0.15 mg mL^−1^) was mixed with 1.0 mL of Britton-Robinson (BR) buffer (pH 7) into 5-mL volumetric flask, then 1 mL of working solution of MOD was added to obtain the final concentration range (50 – 700 ng mL^−1^). The resulted mixture completed by ultra-pure distilled water till the marked volume. The fluorescence intensity was measured at λ_em_ 515 nm after 10 min (excitation 445 nm).

### Estimation in Pharmaceutical Product and Content Uniformity Test

Ten Bravamax^®^ tablets (200.0 mg/tablet) were weighed, crushed finally and thoroughly mixed. Then, an amount equivalent to 10 mg MOD was transferred into a volumetric flask and then dissolved into 50 mL of methanol. The solution was sonicated about 20 min followed by filtration, then volume was completed to 100 mL with methanol to get concentration of 100 µg mL^−1^.

For content uniformity test [19–21], each tablet of Bravamax^®^ drug was individually weighed and finally powdered. An amount equivalent to 10 mg from the powder were dissolved into 50 mL methanol with sonication for 20 min followed by filtration to remove undissolved excipients then volume completed to 100 mL methanol to get concentration of 100 µg mL^−1^. Then the analytical procedure was followed.

### Preparation of Spiked Plasma

Into a centrifuge tube, 1.0 mL of human plasma was spiked with adequate amount of MOD solution. then furtherly 1 mL of methanol were added as protein precipitating agent [22, 23]. The mixture was vortexed for 30 s and then completed to 10 mL. The mixture was centrifuged for 30 min (3500 rpm), after that 1.0 mL of supernatant was used in analytical procedure.

### Preparation of Real Sample

Real human plasma samples were conducted in accordance with the responsible committee's ethical guidelines and the 2008 revision of the 1975 Helsinki Declaration. BRAVAMAX^®^ tablets (200.0 mg/tablet) were administrated as single oral dose by 5 healthy volunteers. The blood samples were collected after time intervals (0.25, 0.5, ……to 20 h) into heparinized tubes. The blood samples were centrifuged at 5000 rpm for 30 min to separate the plasma. 1.0 mL of the plasma was mixed with 1 ml of methanol as protein precipitating agent. Then the centrifugation was carried out at 3500 rpm for 30 min to separate the supernatant. The supernatant was utilized in analytical procedure.

## Results and Discussion

### Morphological Characteristics of the Quantum Dots

The surface morphology of N@CQDs was studied using high-resolution transmission electron microscope (HR-TEM). The size of N@CQDs was found to be 2.0 nm ± 0.21. Figure 1b

Dynamic light scattering (DLS) was carried out for particle size confirmation. The size was found to be 2.5 nm, which agrees with HR-TEM image. Fig. S1 (Supplementary Materials). Besides, the powder X-ray diffraction (PXRD) image was utilized to study the formation of N@CQDs, the peak presented at 24.60^o^ is a diagnostic peak of carbon dots [13, 15] as seen in Fig. S2.

As shown in Fig. S3, the energy dispersive X-ray spectrometer (EDX) was carried out to check the presence of (C, N and O) elements. The spectrum shows the presence C, N, and O elements. The function groups formation on the surface of N@CQDs were examined using FTIR spectroscopy Fig. S4. The FTIR peaks appear at 3410 cm^−1^ and 2900 cm^−1^ corresponding to (-NH, -OH) and 2900 cm^−1^–CH groups respectively. The peaks at 1690 and 1560 cm^−1^ correspond to –C = O and –C = C groups. In addition, the peaks at 1556 and 1293 cm^−1^ refer to N–O and C–O stretching Fig. S4.

Furthermore, X-Ray photoelectron spectroscopy (XPS) was carried out for the elemental analysis. The XPS peaks of N@CQDs were observed as three characteristic strong peaks at 284.9, 395.6, and 538.5 eV corresponding to C 1 s, N 1 s, and O 1 s, respectively. It signifies that C-dots are formed from O (43.00%), C (37.89%), and N (19.11%). Fig. S5a.

The results refer to formation of N (19.11%) with percent higher than previously reported methods (6.88%, 9.15%) [24] due to surface passivation of carbon dots particles. In C 1 s spectrum (Fig. S5b) there are four peaks were observed at 284.5, 285.2, 286.4, and 288.5 eV, due to presence C = C, C-N, C-O, and C = O groups, respectively. For N 1 s spectrum, 2 peaks are observed at 399.2 eV and 400.8 eV, produced due to presence of C-N and N–H as shown in Fig. S5c [25].

For O 1 s spectrum has two peaks for C–OH, C–O–C and C = O at 531.4 eV and 532.6 eV (Fig. S5d) [13, 26]. The morphological characters of quantum dots demonstrate the structure of N@CQDs contain numerous of function groups, which interpretate the interaction between MOD and N@CQDs via electrostatic interaction and hydrogen bonding.

The quantum yield (QY) of N@QDs was studied via single point method using the following equation:

$${Q}_{NCQDs}={Q}_{Quinin} \times \frac{{F}_{NCQDs}}{{F}_{Quinin}} \times \frac{{A}_{st}}{{A}_{NCQDs}} \times \frac{{\eta }^{2} (NCQDs)}{ {\eta }^{2} (Quinin)}$$

Q is the quantum yield while F is integrated fluorescence.

The proposed method provides high quantum yield due to reducing the sizes of N@CQDs (2.0 nm) would increase the quantum yields by creating more optical effects via increasing number of function groups on the surface of quantum dots. [27, 28] Furthermore, *Eruca Sativa* leaves are rich with various components and vitamins as carbohydrates, sugar, fibers, vitamin A, vitamin B_1_, riboflavin and folic acid which led to numerous and varying function groups during quantum dots synthesis. The quantum yield of amine quantum dots was found to be = 41.39%.

### Optical Characters of the Green Synthesized Quantum Dots

**Fig. 2:**
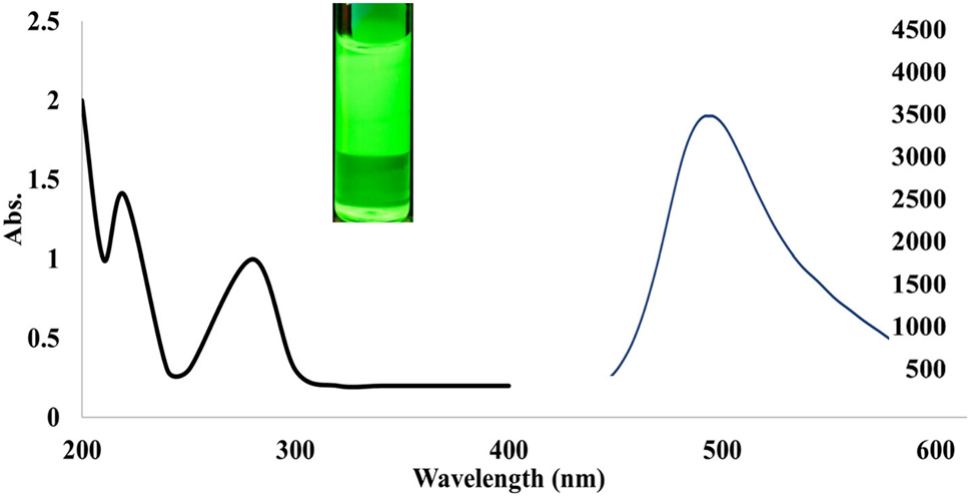
Optical characters of the green synthesized N@CQD

The green N@CQDs showed two UV spectra at 229 and 296 nm as in Fig. 2. These peaks were referred to π-π* electronic transition of C = C and n-π* electronic transition of C = O related to amine carbon dots surface. Moreover, N@CQDs produce emission peak at 515 nm (λ_ex_ 445 nm), which indicate optical characters of carbon quantum dots Fig. 2.

**Fig. 3:**
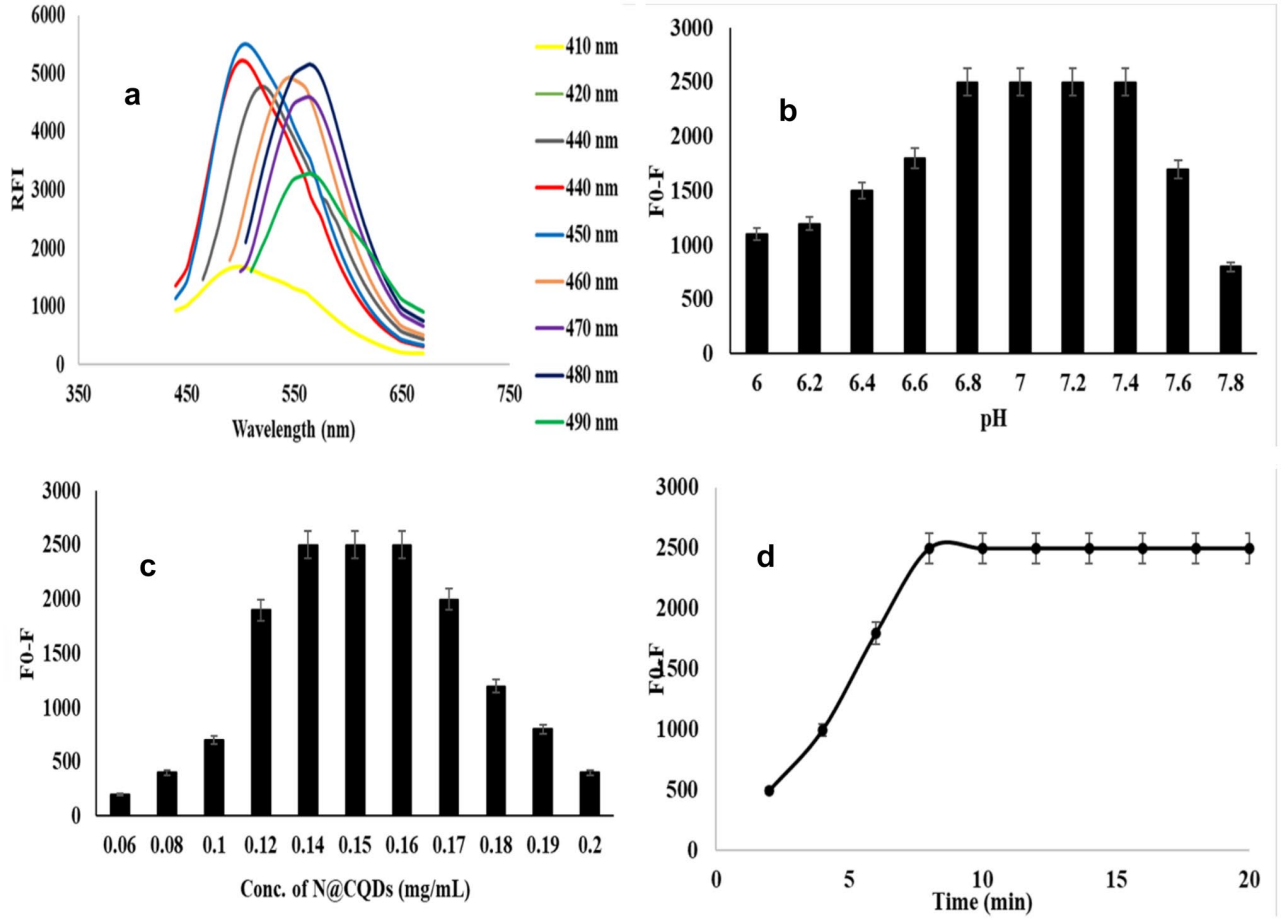
**a** Excitation dependent emission curves for N@CQDs, **b** Effect of pH, **c** Effect N@CQDs concentrations and **d** Reaction time of MOD (300 ng mL ^−1^) with N@CQDs

The relative fluorescence spectra of N@CQ-dots were scanned with increased excitation wavelengths from 410 to 490 nm, the increasing excitation wavelengths led to a red shift in the emission spectra followed by a decrease in RFI, confirming carbon dots excitation-dependent emission Fig. 3a.

Moreover, the effect of temperature in the range of (25 – 60 ^o^ C) on relative fluorescence intensity (RFI) of N@CQDs was studied. Increasing temperature beyond 25 ^o^ C declined the fluorescence intensity [13, 15].

### Optimization of the Methodology

The pH effect on N@CQD RFI in the presence and absence of MOD was investigated, due to the presence of various function groups, N@CQDs were observed to quench steadily in the pH range of 6.8 to 7.4 and raising the pH to 7.5 caused an unstable decrease in RFI. So, the ideal pH was determined to be 7 Fig. 3b.

Furthermore, Various concentrations of N@CQDs were tested during the reaction with MOD (300 ng mL^−1^), it was found that 0.15 mg mL^−1^ (1.0 mL) produce most stable quenching Fig. 3c.

The time of the reaction of MOD with N@CQDs in pure form was studied at different time intervals ranging from 0 to 20 min. The maximum stable quenched was observed within 10 min Fig. 3d.

### Reaction Validation of MOD with N@CQDs Validation

**Fig. 4:**
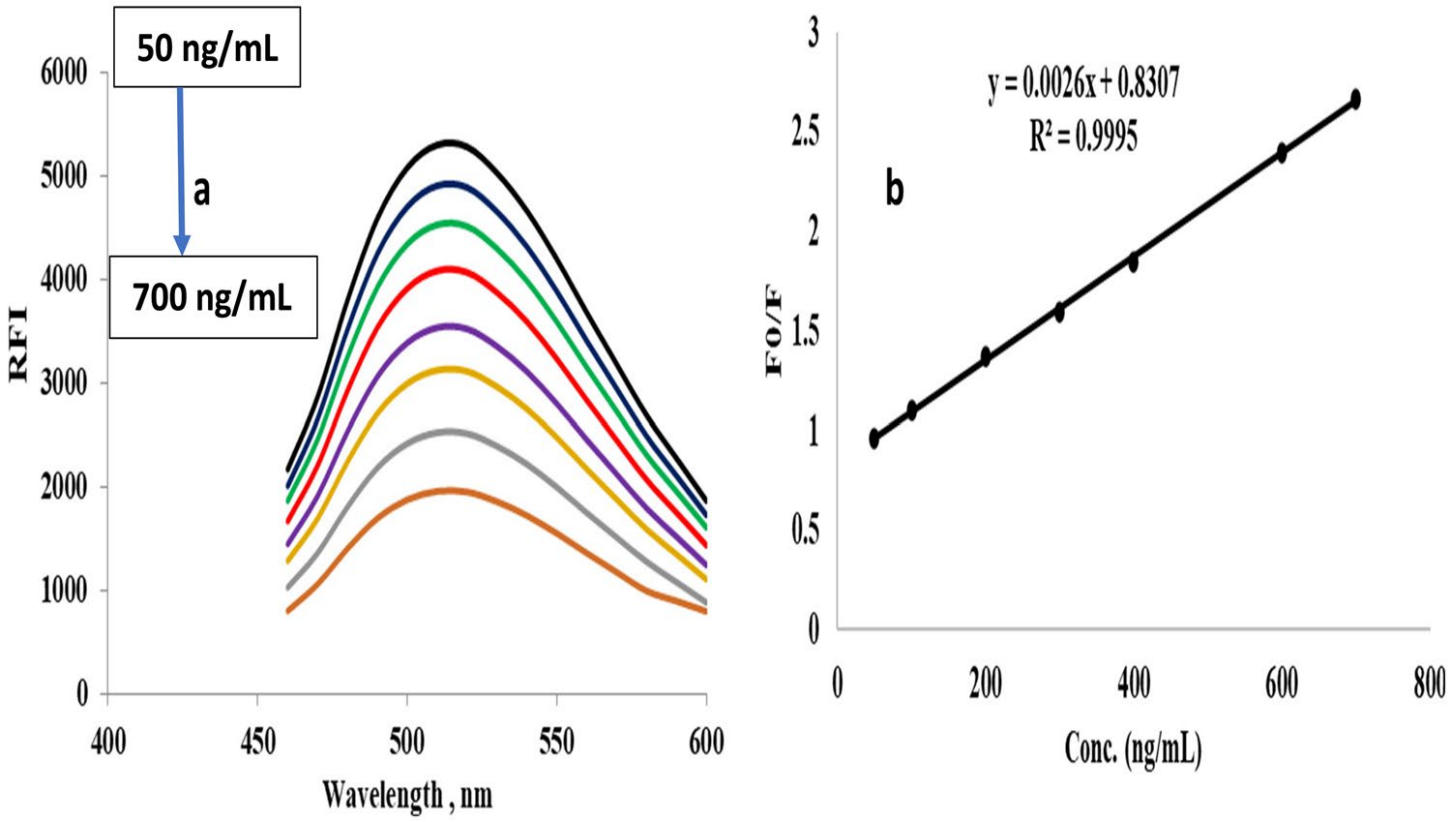
**a** Reaction of N@CQDs with MOD at different concentrations, and **b** Stern–Volmer curve for MOD with N@CQDs

The presented approach for the reaction of modafinil in the presence of N@CQDs was validated at optimum conditions using International Conference of Harmonization (ICH) and US-FDA rules [29, 30]. The green synthesized N@CQDs fluoresence was quenched with inceasing modafinil concentration at 515 nm (excitation at 445 nm) Fig. 4a.

The regression equation was found to be Y = 0.0026x + 0.8307 using the Stern–Volmer equation, good linearity was established within the linear range of 50–700 ng mL^−1^, with a correlation coefficient is 0.9995 Fig. 4b.

$${\mathrm{F}}_{0}/\mathrm{F }= 1+\mathrm{Ksv }[\mathrm{Q}]$$

where, F_0_ and F are the fluorescence efficiencies of the N@CQDs in the absence and presence of the MOD, respectively. [Q] is MOD concentration and Ksv is the Stern–Volmer constant.

The presented is ultra-sensitive one with limit of detection (LOD) value 14.85 ng mL^−1^ and limit of quantitation (LOQ) 45.0 ng mL^−1^ as seen in Table 1.

**Table 1:** Quantification parameters for estimation of MOD using N@CQDs

Parameter	Results
λ_ex_ (nm)	445
λ_em_(nm)	515
Concentration range (ng mL^−1^)	50 –700
Determination coefficient (r^2^)	0.9995
Slope	0.0026
Intercept	0.8307
SD the intercept (Sa)	0.009
LOD (ng mL^−1^)	14.85
LOQ (ng mL^−1^)	45.0

*LOD* lower limit of detection

*LOQ* lower limit of quantitation


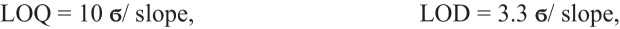


 is standard deviation.

Different concentrations form MOD within the calibration range (50, 100.0, 300.0, 500.0, and 700.0 ng mL^−1^) were employed to examine the N@CQDs method's accuracy. According to Table 2, the RSD values ranged from 0.40 to 1.12, and the recovery percentage ranged from 99.68 to 102.11%. The reproducibility of the presented method was further examined using three replicates of each concentration (100.0, 300.0, and 500.0 ng mL^−1^). According to Table 2, the RSD value was (0.16 to 1.11), showing high reproducibility of N@CQDs method.

**Table 2:** Accuracy and precision of the proposed method for determination of MOD in pure form

Sample number	Taken Conc (ng mL^−1^)	Found Conc (ng mL^−1^)	% Recovery ^*^ ± RSD
1	50.0	50.34	100.68 ± 1.00
2	100.0	102.11	102.11 ± 1.12
3	300.0	306.18	102.06 ± 0.74
4	500.0	498.40	99.68 ± 0.60
5	700.0	710.41	101.48 ± 0.40
Intra-day precision	100.0	101.83	101.83 ± 0.64
	300.0	305.54	101.84 ± 0.80
	500.0	503.10	100. 62 ± 0.16
Inter-day precision	100.0	101.09	101.09 ± 0.42
	300.0	301.35	100.45 ± 1.05
	500.0	502.44	100.48 ± 1.11

^*^: Average of three determinations. *RSD* Relative standard deviation

A bio-analytical validation of the reaction of MOD with N@CQDs in human plasma was examined via three concentrations (100, 300, and 600 ng mL-1) according to US-FDA recommendation. The RSD value ranged from 1.79 to 2.33, as reported in Table 3, the results indicate to high accuracy of N@CQDs method in human plasma.

**Table 3:** Bio-analytical validations of the N@CQDs study for determining MOD concentration in human plasma

	Intra-day assay(n = 6)			Inter-day assay(n = 18)		
Conc (ng mL^−1^)	Found (ng mL^−1^)	Accuracy (%)	Precision (CV %)	Found (ng mL^−1^)	Accuracy (%)	Precision (CV %)
100	97.60	97.60	2.33	96.80	96.80	1.88
300	290.14	96.71	2.25	288.53	96.17	2.10
600	586.50	97.75	1.79	584.11	97.35	1.90

Furthermore, the robustness of the green synthesized N@CQDS was employed by making slight adjustments to the analytical procedure's parameters. As can be seen in Table 4, there was no significant effect for a slight modification in the technique variables.

**Table 4:** Robustness of the reaction of MOD (300 ng mL^−1^) with N@CQDs

Variations	
	% Recovery ^a^ ± RSD
Optimum condition	101.80 ± 0.30
1- Value of pH (BR buffer)	
6.8	99.91 ± 1.26
7.2	99.84 ± 1.14
2- Volume of buffer (mL)	
0.75	99.66 ± 1.40
1.25	99.80 ± 0.71
3- N@CQDs concentration (mg mL^−1^)	
0.14	99.83 ± 0.94
0.16	99.81 ± 0.52
4- Reaction time (min)	
8	99.97 ± 0.99
12	99.94 ± 0.90

^a^Mean of six determinations

In addition to the stability of modafinil in human plasma was examined using N@CQDs, as shown in Table 5. The stability was studied using three levels: low-quality control (LQC), medium-quality control (MQC) and high-quality samples (HQC) under different conditions. The outcomes refer to that modafinil is stable in human plasma under different condition. Table 5.

**Table 5:** Stability and matrix effect of modafinil in human plasma

Conditions			
Concentrations	LQC 70 ng mL^−1^	MQC 300 ng mL^−1^	HQC 600 ng mL^−1^
Three Freeze–thaw cycle stability (-24 °C)	97.44 ± 2.24	96. 94 ± 2.44	97.10 ± 0.91
Long-term stability (1 months at -24 °C)	96.98 ± 1.60	97.85 ± 2.11	97.23 ± 2.05
Short-term stability (12 h at -24 °C)	97.10 ± 1.82	96.88 ± 1.94	97.21 ± 2.17
Post-preparative stability (6 h at room temperature 25 °C)	97.76 ± 1.52	97.22 ± 1.44	97.13 ± 2.02
Post-preparative stability (12 h at room temperature 25 °C)	96.89 ± 2.05	97.13 ± 1.80	96.40 ± 1.29

To further assess the accuracy and precision of the incurred plasma sample of modafinil incurred sample reanalysis (ISR) was performed. The range of values between the original samples and the incurred samples was found between 3.35 and 5.84%, according to Table 6.

**Table 6:** Incurred sample reanalysis for estimation of MOD using the proposed method

Sample	Intial concentration* (ng mL^−1^) ± SD	Incurred concentration* (ng mL^−1^) ± SD	% Deviation
1	470.0 ± 1.73	450.0 ± 2.31	- 4.25
2	460.60 ± 2.40	445.13 ± 1.59	- 3.35
3	480.19 ± 1.16	452.11 ± 2.72	- 5.84

^*^: Mean of three determinations

The selectivity of N@CQDs was performed to evaluate the excipient interference. Talc, starch, mannitol, magnesium stearate, lactose, sodium chloride, and other excipients were evaluated alongside MOD. The findings demonstrated that the excipients had no effect, demonstrating the excellent selectivity of the indicated approaches Table 7.

**Table 7:** Effect of different excipients for estimation of MOD using the proposed method

	Recovery* ± RSD
Mannitol	101.61 ± 1.33
Talc	99.84 ± 0.40
Starch	102.00 ± 0.69
Lactose	101.30 ± 0.77
Magnesium stearate	101.31 ± 0.40
Sodium chloride	99.66 ± 0.34

^*^: Mean of three determinations

### Suggested Reaction Mechanism of the Proposed Method

The reaction mechanism between modafinil and N@CQDs was interpretated with Stern–Volmer equation as:

F_0_/F = 1 + Ksv [Q], The quenching mechanism is dynamic clearly indicated by the linearity of the Stern–Volmer figure. modafinil interacts with excited N@QDs, causing energy/electron transfer and dimming of the quantum dots' fluorescence. The Stern–Volmer model perfectly describes this process Fig. 4b.

In addition to allowing for the creation of hydrogen bonds and electrostatic attraction between MOD and nitrogen dopped carbon dots, the existence of numerous function groups in MOD allow for these other phenomena [13, 31].

### Applications of N@CQDs in Human Plasma

The N@CQDs method's ultra-sensitivity enables the detection of MOD in human plasma that has been spiked. The investigated procedure at six distinct concentration levels applied was found to have a recovery percentage that ranged from 95.25% to 98.34%. The SD of the data was in the range of 0.97 to 2.17, which is within the permissible bounds of the analytical method variability resulting from various matrix effects as indicated in Table 8.

**Table 8:** Application of the N@CQDs method for determination of modafinil in spiked human plasma

Added conc (ng mL^−1^)	Found Conc (ng mL^−1^)	% Recovery ^*^ ± RSD
50	48.75	97.50 ± 1.24
100	98.34	98.34 ± 0.97
200	190.50	95.25 ± 1.80
400	387.40	96.85 ± 2.17
500	482.41	96. 48 ± 2.05
700	685.51	97.93 ± 1.88

^*^Average of six determinations

Modafinil is neuro-stimulants antinarcoleptic drug that help with the post-COVID neurological syndrome. The pharmacokinetic of MOD was investigated via healthy human volunteers, the maximum plasma concentration was found to be C_max_ was determined to be 4.15 ± 0.67 µg mL^−1^, and t_max_ is 2.0 ± 0.55 h, t_1/2_ equal to 12.30 h and area under curve was found to be (AUC_0-ꝏ_) 60.33 ± 10.4 µg.h mL^−1^. The results were displayed in Table 9 and were closely related to the approach that had previously been reported [32].

**Table 9:** Pharmacokinetic study for estimation of modafinil using N@CQDs

Time (h)	Found Conc (µg mL^−1^)	Parameters	Results
0.5	0.82 ± 0.50	C_max_ **(µg mL**^**−1**^**)**	4.15 ± 0.67
1	1.60 ± 1.32	t_max_ **(h)**	2.0 ± 0.55
2	4.15 ± 0.67	t _½_ **(h)**	12.30 ± 0.51
3	4.00 ± 2.21	AUC **(µg·h mL**^**−1**^**)**	60.33 ± 10.14
5	3.84 ± 1.11		
7	3.60 ± 3.76		
9	3.57 ± 1.60		
11	2.10 ± 1.60		
12	2.00 ± 0.55		
14	1.60 ± 0.70		
16	1.37 ± 0.79		
18	1.22 ± 0.40		
20	1.18 ± 0.33		

### Applications in Pharmaceutical Dosage form and Content Uniformity Test

The recommended method (N@CQDs) was used with effectiveness for quantifying MOD in commercial tablets (BRAVAMAX^®^ 200 mg). The percentage of recovery ± SD was discovered to be 102.33 ± 1.05 as compared with reported method [9] (99.44 ± 1.09). Additionally, the results of the t-test and F-test were found to be 1.61 and 2.91, respectively. At 95% confidence, the results did not significantly differ between the proposed and reported method.

Each unit in a batch should have a drug substance composition that falls within a specific range around the label claim in to guarantee the uniformity of dosing units. For MOD, the method was ideally suited for content uniformity testing, which is a time-consuming process when using conventional assay techniques. This was because the proposed method has a high sensitivity and can quickly and accurately measure the fluorescence intensity of a single tablet extract. The test's phases were implemented in accordance with USP protocol [33]. When the acceptance value (AV) was calculated, it was discovered that it was lower than the permitted maximum acceptance value (L1). As indicated in Table 10, the outcomes showed good medication consistency.

**Table 10:** Content uniformity for estimation of MOD in pharmaceutical product using N@CQDs

Tablet No	% Labeled claim
	Bravamax® tablets (200 mg modafinil/ tablet)
1	101.66
2	101.90
3	102.09
4	100.16
5	99.68
6	100.98
7	101.74
8	101.19
9	99.99
10	101.12
Mean	101.05
SD	0.71
RSD	0.70
Acceptance value (AV)*	1.70
Max. allowed AV (L1)*	15

^*^ Acceptance value = 2.4 × SD

### Assessment of the Greenness of the Proposed Method Versus Reported Method

Several assessment tools have been recently reported for evaluation of the ecological impacts of the analytical methodologies. The assessment of analytical methods helps in reduction of environmental pollution generated by such processes. For instance, an average of 0.5L of organic waste is generated daily from a conventional HPLC system [34] therefore, the greenness assessment became a must do evaluation. Since the proposed method is suitable for application in determining MOD in pharmaceutical dosage forms as well as plasma samples, the method is to be compared to another previously reported method [2]. As seen in Table 11, two green assessments were utilized for estimation the greenness of the proposed method GAPI and AGREE methods [35–37]. The results in Table 11 refer to high greenness effect of the proposed method (N@CQDs) which agree with US-climate change conference.

**Table 11:**
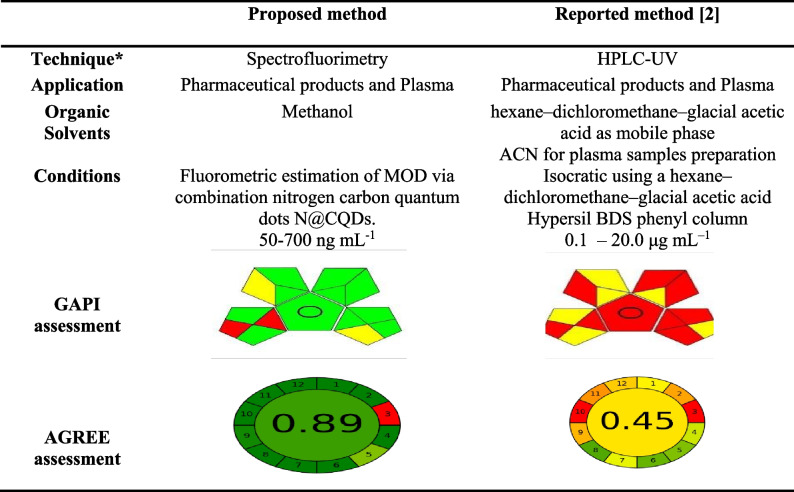
Comparison between the proposed and reported method for determination of MOD under different applications

## Conclusion

The presented study provides novel, simple, environmentally friendly, highly sensitive, economical, and quick approach for the synthesis of N@CQDs as fluorescent probes from green source. A one-pot, low-energy, chemical-free carbonization utilizing a conventional microwave has been used to quickly develop the N@CQDs. The suggested approach was validated, and bio analytically validated using ICH and US-FDA requirements. In both a content uniformity test and a pharmacokinetic investigation, this sensor was successfully used to determine MOD. As a result, this straightforward and label-free sensing platform was used as the fluorescence-based assessment of the target analyte without the need for chemical derivatization or the extensive reaction times that are required by previously published methods.

## Supplementary Information

Below is the link to the electronic supplementary material.

Supplementary file1 (DOCX 661 KB)

## Data Availability

All data generated or analyzed during this study are included in this published article (and its supplementary information files).
