# Supplementary material for: Rapid One-Pot Microwave Assisted Green Synthesis Nitrogen Doped Carbon Quantum Dots as Fluorescent Precursor for Estimation of Modafinil as Post-Covid Neurological Drug in Human Plasma with Greenness Assessments
Source: J Fluoresc. 2022 Dec 28;33(3):1101–10. doi: 10.1007/s10895-022-03128-5 (PMC9795147; doi:10.1007/s10895-022-03128-5)
Supplement: Supplementary file 1 — Supplementary file1 (DOCX 661 KB) [file 10895_2022_3128_MOESM1_ESM.docx]

# Rapid one-pot microwave assisted green synthesis nitrogen doped carbon quantum dots as fluorescent precursor for estimation of modafinil as post-covid neurological drug in human plasma with greenness assessments

Baher I. Salman ^a^, Ahmed I. Hassan ^a^, [Yasser](https://www.nature.com/articles/s41598-022-10779-8#auth-Mohamed_A_-Abdel_Lateef) F. Hassan ^a^, Roshdy E. Saraya ^b^ and Hany A. Batakoushy ^c^

^a^ Pharmaceutical Analytical Chemistry Department, Faculty of Pharmacy, Al-Azhar University, Assiut branch, Assiut, 71524, Egypt.

^b^ Pharmaceutical Analytical Chemistry Department, Faculty of Pharmacy, Port Said University, Port Said 42511, Egypt.

^c^ Pharmaceutical Analytical Chemistry Department, Faculty of Pharmacy, Menoufia University, Shebin Elkom, 32511, Egypt.

# * Corresponding author: Baher I. Salman

Email: [bahersalman@azhar.edu.eg](mailto:bahersalman@azhar.edu.eg) , [bahersalman2013@yahoo.com](mailto:bahersalman2013@yahoo.com)

Tel. +201099031345

**
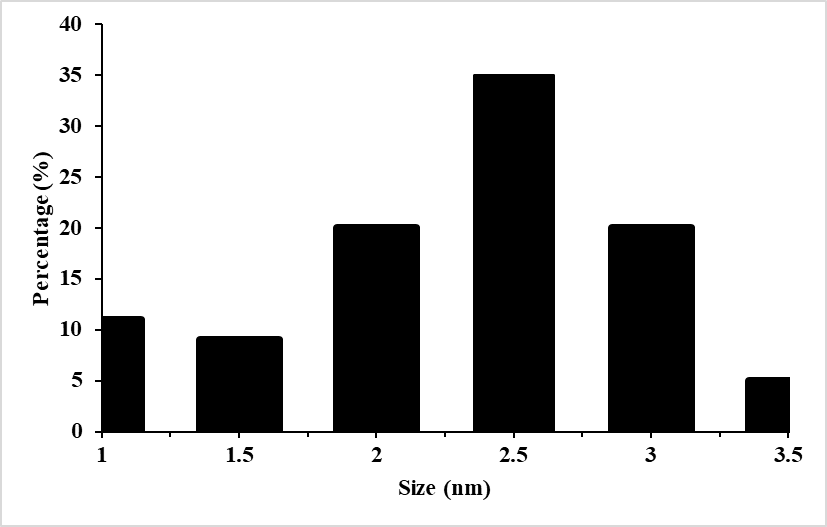
**

**Fig. S1** DLS image for synthesized green N@CQDs.

**
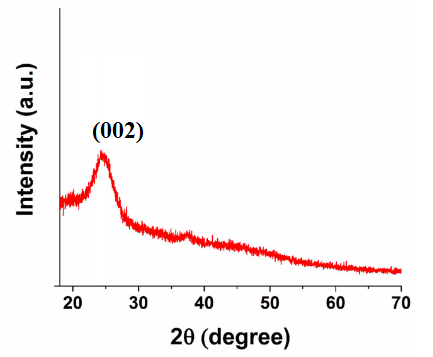
**

**Fig. S2** PXRD curve for N@CQDs.


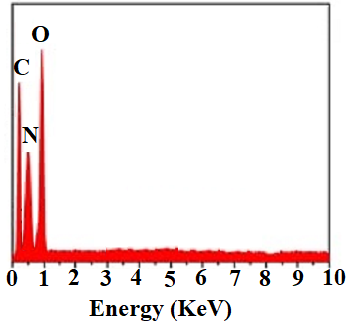


**Fig. S3** EDX for characterization of CQDs


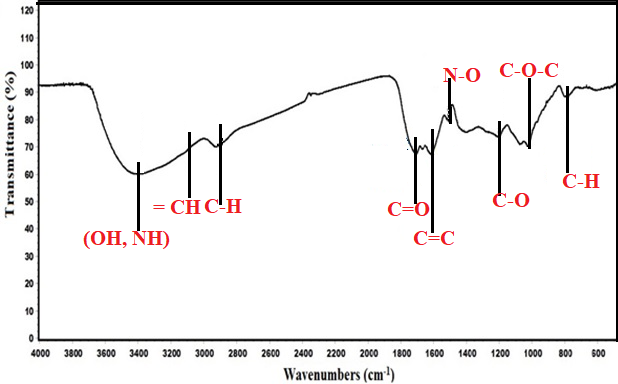


**Fig. S4** FTIR spectroscopy for characterization of N@CQDs.


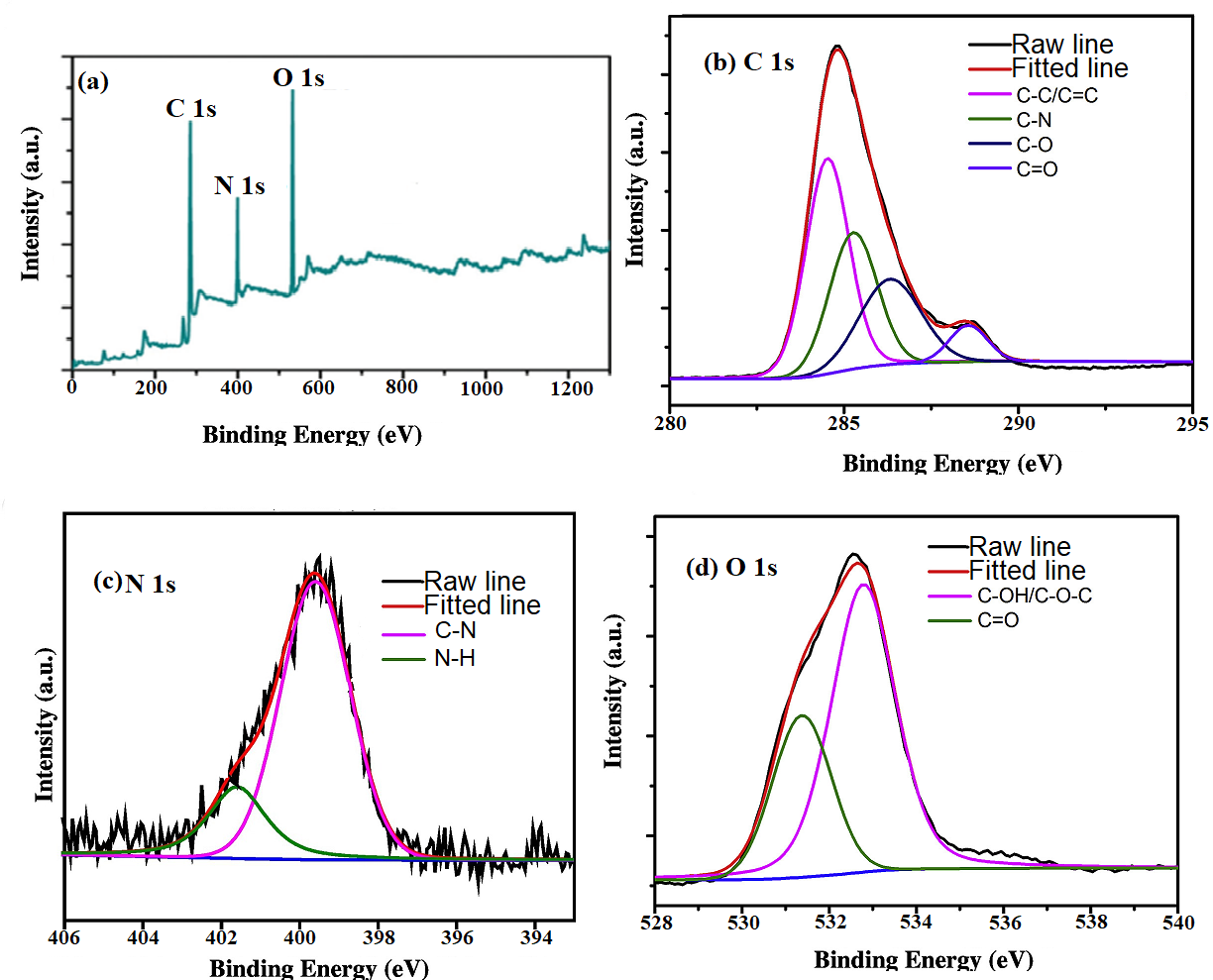


**Fig. S5** **a)** XPS spectrum, **b)** C 1s spectrum **c)** N 1s spectrum and **d)** O 1s spectrum for element analysis of N@CQDs.
